# Using internet-assisted geocoding of 1940 census addresses to reconstruct enumeration districts for use with redlining and longitudinal health datasets

**DOI:** 10.1371/journal.pgph.0004067

**Published:** 2025-01-15

**Authors:** Shuo Jim Huang, Michel Boudreaux, Kellee White Whilby, Rozalina G. McCoy, Neil Jay Sehgal

**Affiliations:** 1 University of Maryland Institute for Health Computing, North Bethesda, Maryland, United States of America; 2 Department of Health Policy and Management, University of Maryland School of Public Health, College Park, Maryland, United States of America; 3 Division of Endocrinology, Diabetes, & Nutrition, Department of Medicine, University of Maryland School of Medicine, Baltimore, Maryland, United States of America; 4 Division of Gerontology, Department of Epidemiology and Public Health, University of Maryland School of Medicine, Baltimore, Maryland, United States of America; 5 Department of Health Systems and Population Health, University of Washington School of Public Health, Seattle, Washington, United States of America; Merck & Co., Inc., UNITED STATES OF AMERICA

## Abstract

Many historical administrative documents, such as the 1940 census, have been digitized and thus could be merged with geographic data. Merged data could reveal social determinants of health, health and social policy milieu, life course events, and selection effects otherwise masked in longitudinal datasets. However, most exact boundaries of 1940 census enumeration districts have not yet been georeferenced. These exact boundaries could aid in analysis of redlining and other geographic and social contextual factors important for health outcomes today. Our objective is to locate and map a large set of 1940 enumeration districts. We use online resources and algorithmic solutions to locate and georeference unknown 1940 enumeration districts. We geocode addresses using the OpenCage API and construct “virtual” enumeration districts by using a convex hull algorithm on those geocoded addresses. We also merge in Home Owners’ Loan Corporation (HOLC) redlining maps from the 1930s to demonstrate how 1940 enumeration districts could be used in future work to examine the association between historic redlining and current health. We geocode 7,228,656 1940 census addresses from the largest 191 US cities in 1940 that contained 84% of the 1940 US urban population from the Geographic Reference File and construct 34,472 virtual enumeration districts in areas that had HOLC redlining maps. 18,340 virtual enumeration districts were previously unmapped, covering cities containing an additional 40% of the 1940 US urban population. Where virtual enumeration districts match with previously mapped districts, 96.8% of paired districts share HOLC redlining categorization. Researchers can use algorithmic methods to quickly process, geocode, merge, and analyze large scale repositories of historical documents that provide important data on social determinants of health. These 1940 enumeration district maps could be used with studies such as the Health and Retirement Study, Panel Study for Income Dynamics, and Wisconsin Longitudinal Study.

## Introduction

### Need for historical geocoded data

A large and diverse literature shows long-run outcomes in health, economic attainment, and other measures of social well-being are heavily influenced by place effects in early life, driven by policy, environmental, and socio-economic factors [1–8]. A separate but complementary literature studies the persistence of social outcomes within geographic areas and the reasons for such persistence [9–19]. These literatures require data describing populations and their geographic location over a long time horizon.

Large quantities of historical documents have been digitized to varying degrees of informational completeness with respect to the original analog documents, and have been made available in open access data portals [20,21]. These documents can provide data for important contextual factors that are critical to understanding structural and social determinants of health that operate throughout the life course both for individuals and populations [21].

The ability to geolocate these historical data allows researchers, advocates, and policy makers to apply geospatial factors and analyses, including areal measures of health determinants such as socio-economic status, housing quality, pollution and other environmental factors, proximity and travel times to healthcare facilities, and access to amenities for physical activity, to name a few [2]. In some cases, historical maps have also been digitized [20]. These can be especially important in the study of racial disparities in healthcare and health outcomes [2], given how past racist policies were entrenched by geography [3,11,20].

Since documents exist in differing states of digitization and informational completeness–anything from simple scans or images of documents to databases of documents that have been fully captioned, georeferenced, coded, and cleaned–researchers can benefit from methods that would allow them to process and merge additional layers of geospatial data with those documents. Geocoding–the process of assigning standardized geographic markers such as latitude and longitude to historical documents and documentary data that can then be mapped–can allow researchers to combine longitudinal and health datasets with a rich matrix of historical, policy, and geographic data [22].

The lowest level of well described and digitized historical geographic boundaries is often county. However, counties encompass heterogeneous, micro-geographic areas [23,24]. The full count decennial censuses can provide such historical sub-county data on populations via enumeration districts [21], but their identification of each enumeration district’s geographic location is incomplete.

We aim to provide a novel method to geocode, reconstruct, and map 1940 census enumeration districts, which can then be used to bridge and map health outcomes data like the Health and Retirement Study with historical and geographic data like redlining datasets. We provide an example of reconstructed 1940 census enumeration districts merged with redlining data, and make them publicly available in a repository.

### Redlining and the 1940 census

Since the public release of georeferenced and digitized Home Owners’ Loan Corporation (HOLC) maps by the Mapping Inequality Project [20], a substantial body of scholarship has examined associations between 1930s redlining and present day health [2]. As part of the New Deal in the 1930s, the Federal government used the HOLC to draw real estate investment security maps [20]. Areas were color-coded or “redlined” to reflect predominant neighborhood race, discouraging lending in majority Black neighborhoods [11]. HOLC used four categories (denoted by a letter and a color) to code areas: A Green “Best”, B Blue “Still Desirable”, C Yellow “Definitely Declining”, and D Red “Hazardous” [20]. Neighborhoods “redlined” [11,20] in the 1930s are still associated with worse financial [10,16,25] and health outcomes [25–35] today. Redlining was likely a proxy for a matrix of discriminatory antecedent conditions, policymaker attitudes, and decades-long policy vectors [11,36]. This century long path dependence [9] masks the potentially differing roles of social policies throughout the 20th and 21st centuries acting on marginalized, primarily Black communities and individuals [37,38], including the impact of lifetime discrimination [3] and of displacement and gentrification [39], and may impact healthcare utilization in the present day [4,40,41]. Thus previous area estimates of health effects associated with redlining [25–35] may poorly capture the full impact of initial conditions from redlining on individuals since it is difficult to disentangle the effects of an area’s population change over a century from the effects of disinvestments in social and built environments using present-day area measures of health effects [35]. Clarifying the policy mechanisms that redlining may have acted through, and connecting health, economic, and utilization outcomes specifically to redlining status close to the 1930s, could allow researchers to examine the role of initial conditions and present day health. Since redlining was a phenomenon operating at the sub-county and sub-city level, we would need data that is available at micro-geographic scales from close to when the maps were drawn in the 1930s.

The 1940 census (the first census taken immediately after redlining) is publicly available on the internet and includes enumeration district identifier data in the form of enumeration district names [21]. Enumeration districts were small geographic units used as census administrative units that were not necessarily coterminous with other geographic divisions [42,43]. Many were hand-drawn using maps most conveniently available to census enumerators, including non-standard maps [42,43]. The 1940 full-count data indicates each individual’s enumeration district. However, the census enumeration districts have not yet been fully geocoded and existing archival maps have not been georeferenced (meaning having standardized geographic features assigned to them). This limits the usefulness of the enumeration district identifiers for geographic analysis. Geocoding the enumeration districts would allow the linking of individuals observed in the 1940 Census with their community context, including their exposure to redlining. Currently, only 40 large US cities have publicly available georeferenced 1940 enumeration districts [43].

191 of the largest US cities in 1940 have publicly available data that could potentially allow us to reconstruct additional enumeration districts. In this study, we reconstruct and map more 1940 enumeration districts than have been mapped before, covering nearly double the 1940 US urban population that was previously covered. These additional mapped enumeration districts will allow researchers to track a group of individuals from their exposure close to when the HOLC drew their redlining maps in the 1930s to those same individuals’ outcomes as elderly adults some fifty to eighty years later in datasets with 1940 census linkages such as the Health and Retirement Study, the Panel Study of Income Dynamics, and the Wisconsin Longitudinal Study [21], rather than tracking only present-day residents of formerly redlined areas. It would also be useful to other researchers working with other health or socioeconomic datasets which have linked 1940 enumeration district data from the full count census but no present way to connect those enumeration districts to geographic factors from either the mid-20th century or today. Additional geographic, contextual data important to health outcomes beyond just HOLC redlining that could be linked might include data on age structure, income, occupation, or other demographic data [21]; existence of and changes in infrastructure and transportation networks [44]; epidemiological studies of disease outbreaks [24]; or maps of historical environmental hazards such as wildfires [45], flooding [46], pollution [17,18,47], or seismological events [48].

## Methods

To create our dataset, we combine data from multiple historical and geographic sources. To construct enumeration districts using address locations, we use the Geographic Reference File [49] and the OpenCage database [50]. For a subset of already defined enumeration districts, we use the city enumeration shapefiles of the Urban Transition Project [43]. For HOLC categories, we use HOLC maps as digitized and georeferenced by the Mapping Inequality Project [20]. Finally, we use the National Historical Geographic Information System’s (NHGIS) dataset of 1940 county boundaries [51]. Our dataset of 1940 enumeration district shapefiles and code to construct the dataset are publicly available in a repository: https://github.com/sjhuang1/1940-Census-Enumeration-Districts.

This research was determined exempt from review by the University of Maryland College Park Institutional Review Board.

### 1940 enumeration districts

The 1940 full-count census data contain the identifier for each participant’s 1940 enumeration district. Enumeration districts were compact geographic areas that one enumerator could cover (by walking in a preset pattern) during the period of the census [42,43]. In urban areas, enumeration districts were particularly dense, and might only cover one or two blocks, or even a single building. Enumeration districts were located within counties, and many cities had their own collection of enumeration districts. Unlike census tracts, enumeration districts could shift drastically both in name identifiers and geographic scope (e.g., size, shape, location) between each census [42,43]. The administrative nature of enumeration districts meant that their boundaries were sometimes informally drawn on maps in non-standardized ways and preservation, digitization, and geocoding of enumeration districts was not prioritized [43].

There does exist a dataset of geocoded 1940 enumeration district geometries from the Urban Transition Project’s publicly available dataset of enumeration districts for 40 large US cities in 1940 as described in Logan and Zhang [43]. Each of the Urban Transition Project’s georeferenced enumeration district polygons is created based on three sources of data: digitized but not georeferenced 1940 enumeration district maps from the National Archives, 1940 enumeration district descriptions from the National Archives, and a set of recreated 1940 street names and locations based on multiple historical data sources. Using this information, the Urban Transition Project then recreates 1940s roads. These roads are then processed by algorithm and manual editing to draw blocks, which are then collated into enumeration districts. The process is resource-intensive, but the results are likely highly precise.

Critically, however, 56% of the US 1940 urban population [52,53] lived in a city whose enumeration districts were not geocoded by the Urban Transition Project. The Urban Transition Project’s dataset also excludes many incorporated near suburbs of most metropolises, where many HOLC green A and blue B graded neighborhoods lay. To solve this problem, we supplement these Urban Transition Project-identified “real” enumeration districts with constructed “virtual” enumeration districts.

### Construction of “virtual” enumeration districts

**Fig 1:**
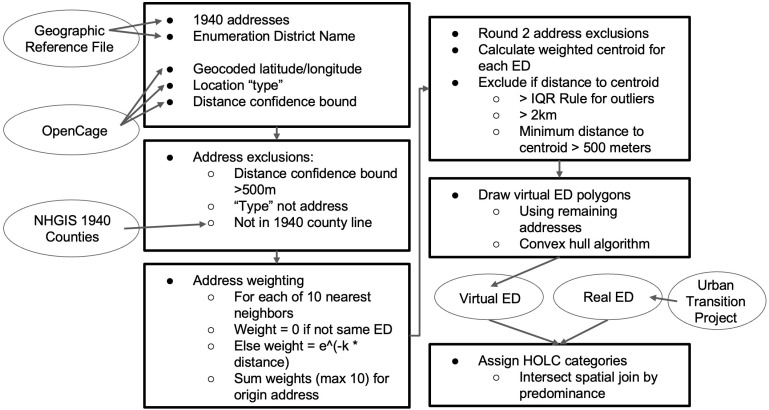
Flowchart of virtual enumeration district construction and assigning HOLC categories.

We geocode a list of 1940 enumeration districts in all 191 cities included in the Urban Transition Geographic Reference File (GRF) (out of 412 total urban places in 1940), covering 84% of the US 1940 urban population. We refer to these as “virtual” enumeration district boundaries because our approach, while providing greater coverage, is more approximate than the boundaries produced by the Urban Transition Project. The general method by which we construct 1940 virtual enumeration districts is to brute force approximate the shape of the enumeration district via bulk address geocoding. We describe the method below and provide a flowchart in Fig 1.

We use the GRF [49], which is a dataset of 191 largest US cities by population in 1940 [49]. A few example rows from the GRF are provided in Figure in S1 Fig. The GRF is constructed from the IPUMS USA version of the 1940 full-count census. For each city, the GRF contains the 1940 address for every enumerated individual from the 1940 census in those 191 cities, along with the full enumeration name. These enumeration district names include the full up-to-4 numeric digit plus the 1 possible alphabetical digit suffix (sometimes with and sometimes without the dash prefix).

Since we are aiming for the geometry of the enumeration district rather than each individual’s specific geographic location within that enumeration district, we assume that the house numbering and street naming scheme of 1940 addresses has not shifted appreciably between 1940 and 2023. We further assume that the geolocation of the same address has not shifted appreciably on the surface of the Earth’s sphere between 1940 and 2023 due to the reconstruction of streets and changes in city layout.

We take advantage of these assumptions to geocode 2023 latitude and longitude to 1940 addresses via OpenCage’s georeferencing database. The OpenCage geocoding algorithm is based in part on OpenStreetMaps. The exact matching and ranking algorithm is not publicly documented but it likely relies on partial string matching and relevance scoring.

While our assumptions may be violated for any given address [43], by focusing on clusters of addresses, we can confidently estimate the location of enumeration districts and construct virtual approximations of those enumeration districts’ polygons. We also then apply exclusion criteria to both trim individual addresses and to exclude malformed virtual enumeration districts using what is known about how enumeration districts were created.

We take each first occurrence of an address in the GRF with its attached enumeration district marker. We use a geocoding application programming interface (API) via Python (packages: opencage [54], asyncio [55]) to query the OpenCage georeferencing database (bounded to US addresses). From the query, we geocode the latitude and longitude of a 2023 address. This provides us with a dataset of enumeration districts, which identifies the latitude and longitude pair for each dwelling within the enumeration district. For 232 of the 7,228,656 observed addresses that returned no result, we corrected malformed addresses by looking at the 1940 full-count census pages [42] and queried OpenCage’s database via its website, or discarded them if there were no matches. 89 of the no result addresses were located in Salt Lake City, UT and 31 in Lincoln, NE.

Since the geocoder may return an imprecise location [56], we retain geocoded locations only with a distance confidence bound of less than 500 meters. We also exclude geocodes if the returned target location is a road or neighborhood rather than an address or address-like feature.

To address known violations of our assumption that correctly named locations have not shifted, we exclude 2023 addresses if they lie outside the bounds of their enumeration districts’ 1940 county lines from the National Historical Geographic Information System (NHGIS).

Undoubtedly, there will be errors for some individual addresses or from some latitude-longitude pairs we associate with those addresses. These errors will appear as location points whose distance to other points in the enumeration district is larger than expected, based on the average distance between points. We address these errors using a multi-step clustering and trimming process to identify a density of points with which to draw the enumeration district. We weight each address by a calculated likelihood to be within the 1940 enumeration district based on its clustering with other addresses. For each address, we calculate a 10 nearest neighbors distance matrix [57] via ball tree algorithm (Python 3.11 packages: sklearn [58], numpy [59], scipy [60], geopandas [61], pandas [62]). We chose 10 nearest neighbors due to limited computational resources available to us. We upweight each address both by the proportion of 10 nearest neighbors that are from the same enumeration district and an exponential decay function on distance (weights are higher the closer a same enumeration district neighbor is). The decay function is:


$$e^{-k\;*\;distance}$$

where *k* is a decay constant set to 5. The weight rapidly degrades as distance increases. In this manner, we generate a weight from 0 to 10 for each address point.

Once we identify the cluster, we trim addresses whose distance to other points in the enumeration district is larger than expected, and thus are likely to be errors. Using QGIS [63], we utilize our weights to plot the weighted centroid of each putative enumeration district and calculate a new distance matrix from that centroid to each address point in the enumeration district. We then exclude address points whose distances-to-centroid lie outside the Interquartile Range Rule for outliers (which is bounded at 1.5 × the interquartile range from the 3rd quartile). Since we are using urban enumeration districts, we also exclude any address points more than 2 km from the centroid. We picked a 2 km radius exclusion zone because mean city-level urban density in the least dense city in 1940 [53] would imply a mean size of 6 sq km per enumeration district, or a 1.4 km radius: we double the area for a valid input in order to be conservative for enumeration districts that would be less dense than the least dense city’s mean density. Finally, to deal with centroids which may have ended up at the midpoint between false clusters, we exclude entire enumeration districts where the minimum distance to the centroid exceeds 500 m (i.e., no addresses found within 1 km diameter of the centroid). These are relatively conservative trims of address points.

**Fig 2:**
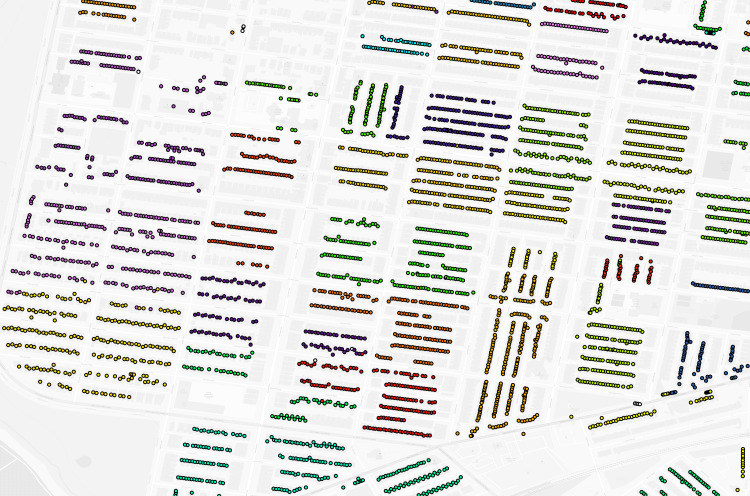
Philadelphia geolocated 1940 addresses by enumeration district (untrimmed). Dot: one 1940 address where shared color indicates membership in the same enumeration district. Background map is a street grid of present day Philadelphia, PA, USA.

**Fig 3:**
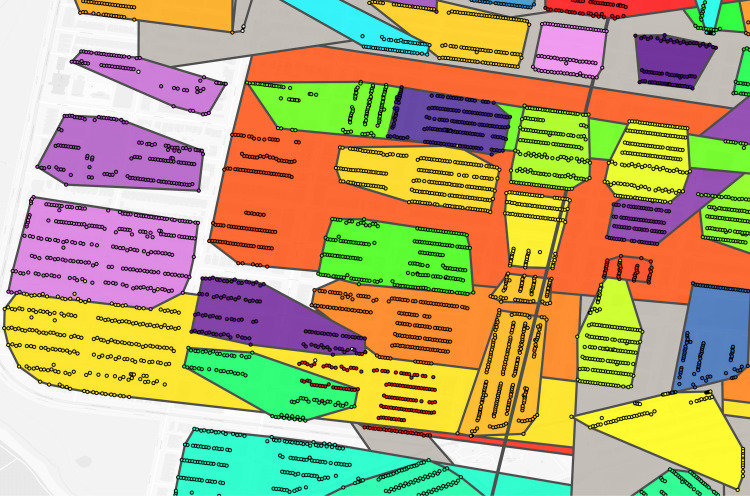
Philadelphia constructed virtual enumeration districts without trimming addresses. Polygon: one virtual enumeration district constructed from untrimmed addresses, dot: one 1940 address. Shared colors between dots and polygons indicate the same enumeration district. Polygons are rendered in descending area size to ensure display of smaller polygons overlapped by larger polygons. Note the influence of erroneous addresses with larger than expected distance to other addresses in the same enumeration district on polygon size.

**Fig 4:**
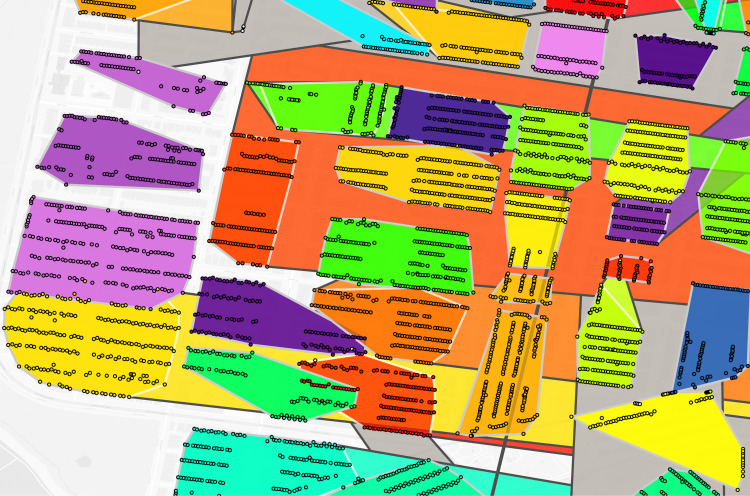
Philadelphia trimmed address virtual enumeration districts overlaid with untrimmed virtual districts. Dot: one 1940 address where shared color refers to enumeration district, white border polygon: one virtual enumeration district constructed from trimmed addresses, black border polygon: one untrimmed virtual enumeration district.

**Fig 5:**
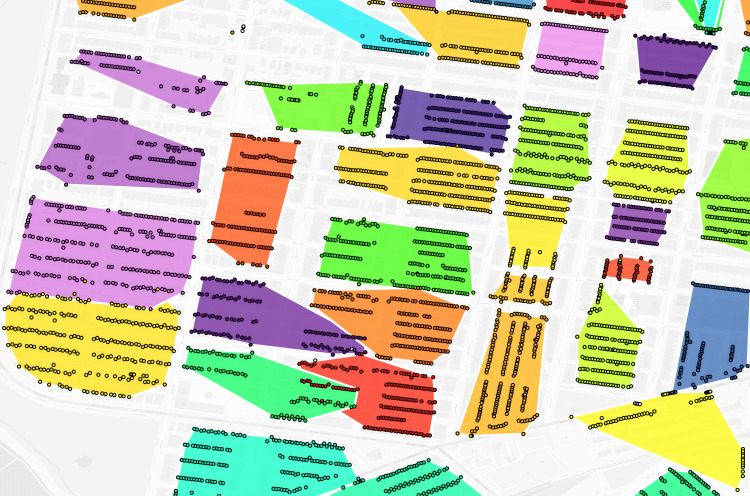
Philadelphia trimmed address virtual enumeration districts overlaid with untrimmed addresses. Dot: one 1940 address where color refers to enumeration district, polygon: one virtual enumeration district constructed from trimmed addresses.

**Fig 6:**
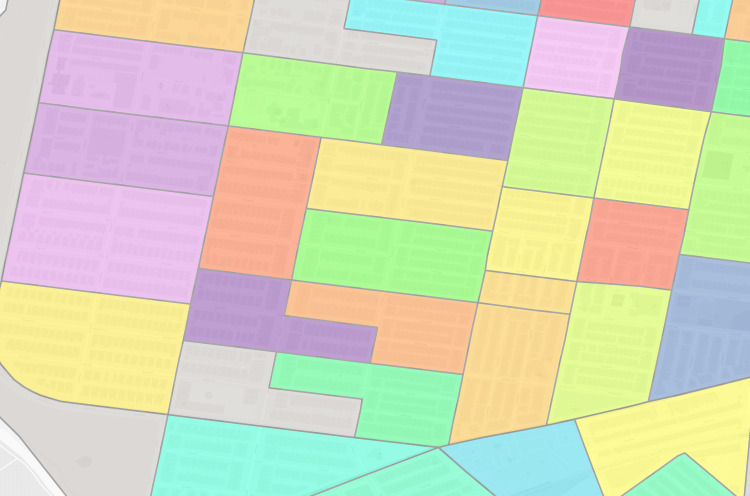
Philadelphia real enumeration districts. Polygon: one real enumeration district from the Urban Transition Project. Black lines are enumeration district boundaries. Polygon color refers to enumeration district. Background map is a street grid of present day Philadelphia, PA, USA (where streets are displayed as white channels).

**Fig 7:**
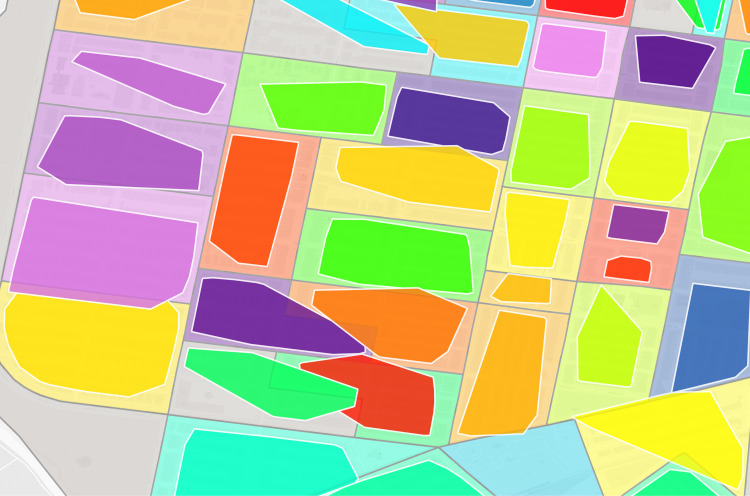
Philadelphia trimmed address virtual enumeration districts overlaid with real enumeration districts. White border darker polygon: one virtual enumeration district constructed from trimmed addresses, black border lighter polygon: one real enumeration district from the Urban Transition Project. Shared colors between the layers indicate the same enumeration district identifier.

**Fig 8:**
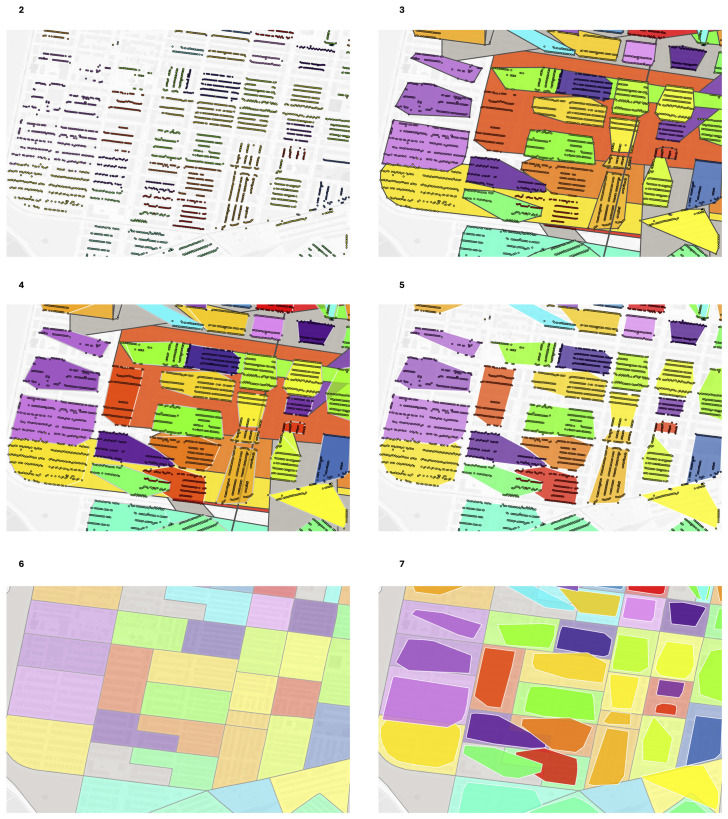
Composite of Figs 2–7 of the same section of Philadelphia. Number above each image refers to source figure. Dot: one 1940 address, black border darker polgyon: one constructed virtual enumeration district without trimming addresses, white border darker polygon: one virtual enumeration district constructed from trimmed addresses, black border lighter polygon: one real enumeration district from the Urban Transition Project. Shared colors between points and different polygon layers indicate the same enumeration district identifier. Background map is a street grid of present day Philadelphia, PA, USA.

A convex hull algorithm draws a polygon bounding a set of points, akin to stretching a rubber band over the outside of a set of pins. Using our final set of trimmed address points projected with a WGS84 Pseudo-Mercator projection, we use an alpha shapes convex hull algorithm via QGIS to draw the minimum bounding polygon around the address points. These polygons are our constructed virtual enumeration districts. We provide an example of the construction process for a section of West Philadelphia in Figs 2–7. We have arranged a composite image of the process in Fig 8.

For each virtual enumeration district, we calculate a geometric centroid (Stata/MP 16.1 geodist package [64]). For geographic locations with both virtual and real enumeration districts, we spatially join the virtual enumeration district’s geometric centroid to the real enumeration district polygon and compare their listed enumeration district names both to aid in pairing and as a final error check. Fig 7 provides an example.

### Assigning HOLC categories

We use an intersect spatial join in QGIS of 1930 HOLC shapefiles to our set of real and virtual enumeration districts. In cases of multiple overlapping HOLC categories, we assign the category by predominance. We do not have HOLC categories for Washington D.C. either because a redlining map was not created or the map was lost [13].

We use a binary weight for assigning HOLC categorization to an enumeration district by whether the enumeration district is “real” or “virtual”. For locations with both a real and a virtual enumeration district polygon, our weight defaults to the HOLC category of the real enumeration district polygon.

## Results

We query and geocode 7,228,656 addresses from the GRF. Our sample covers 42,562 untrimmed urban enumeration districts, including 39,307 urban enumeration districts in cities that also had 1930s HOLC categories. Of those 39,307, we constructed 34,472 trimmed virtual enumeration districts (Table 1). We imported the Urban Transition Project’s real enumeration district boundary shapefiles for 20,967 enumeration districts. 16,132 enumeration districts in our sample have both virtual and real enumeration district boundaries. 18,340 of the virtual enumeration districts, covering cities with 40% of the 1940 urban population, were previously unmapped by the Urban Transition Project. We constructed virtual enumeration districts in 30 cities from the GRF that did not have HOLC categories.

**Table 1:** HOLC Frequency in real and virtual enumeration districts.

HOLC	Real		Virtual	
	**Frequency**	**Percent**	**Frequency**	**Percent**
Total	20967	100	34472	100
A	739	3.5	1176	3.4
B	3271	15.6	5668	16.4
C	8237	39.3	13987	40.6
D	8720	41.6	13701	39.7
Null			20	0.06

Frequencies and percentages for Real and Virtual enumeration districts by HOLC category. Real and virtual categories are not mutually exclusive. HOLC Categories: A: Green “Best”, B: Blue “Still Desirable”, C: Yellow “Defnitely Declining”, D: Red “Hazardous”, Null: mapped by HOLC but not assigned a grade/color.

We found that 96.8% of the 16,132 enumeration districts that had both virtual and real enumeration districts showed no difference in HOLC categorization (Table 2). Only 0.003% of enumeration districts with both virtual and real enumeration districts had HOLC categorizations at least 2 grades apart between the virtual and real enumeration districts.

**Table 2:** Real enumeration district (ED) overlap with virtual enumeration districts.

Real	A	B	C	D	Total
Virtual					
**A**	507	27	8	2	544
**B**	43	2502	99	15	2659
**C**	13	68	6310	134	6525
**D**	3	12	94	6295	6484
**Total**	566	2609	6511	6446	16132

Columns: number of virtual enumeration districts by HOLC category for a given HOLC category in the corresponding real enumeration district. rows: number of real enumeration districts by HOLC category for a given HOLC category in the corresponding virtual enumeration district. Shaded cells display counts of HOLC category concurrence. HOLC Categories: A: Green “Best”, B: Blue “Still Desirable”, C: Yellow“Defnitely Declining”, D: Red “Hazardous”.

**Fig 9:**
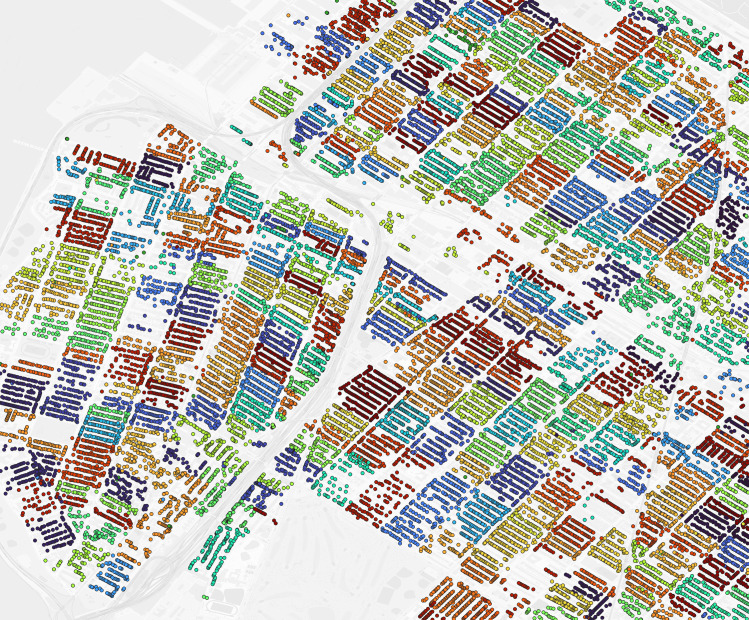
Brooklyn geolocated 1940 addresses by enumeration district. Dot: one 1940 address where shared color refers to enumeration district. Background map is a street grid of present day Brooklyn, NY, USA.

**Fig 10:**
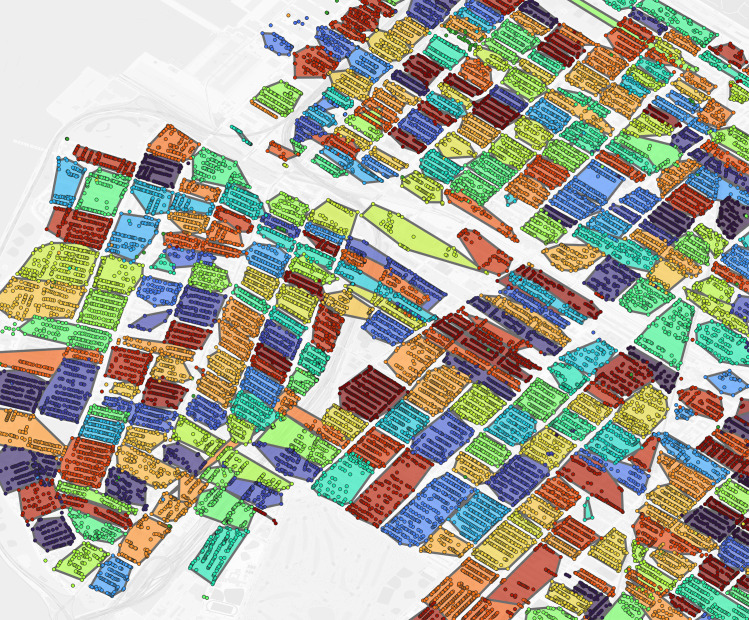
Brooklyn trimmed address virtual enumeration districts overlaid with untrimmed addresses. Dot: one 1940 address where color refers to enumeration district, polygon: one virtual enumeration district constructed from trimmed addresses.

**Fig 11:**
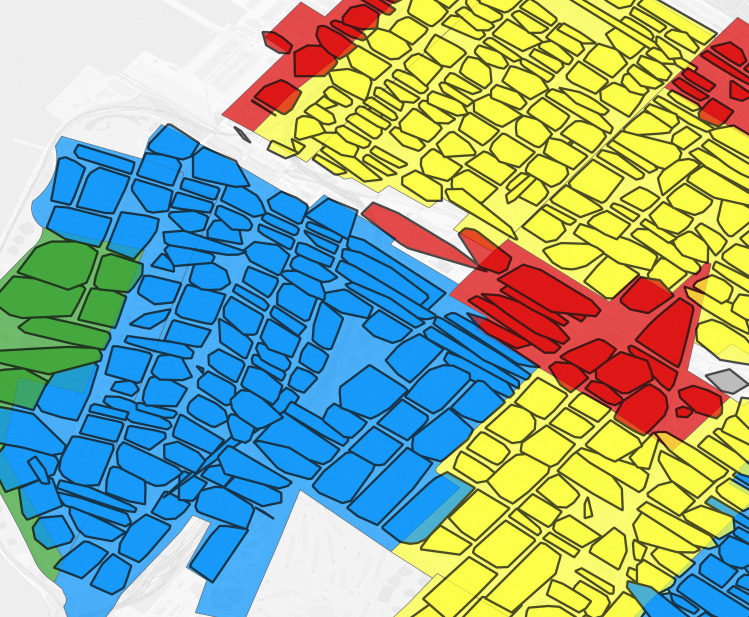
Brooklyn trimmed address virtual enumeration districts tagged by predominance with HOLC redlining categories and overlaid with 1930s HOLC map. Thick black border polygon: one virtual enumeration district where color refers to HOLC categories (green: HOLC A “Best”, blue: HOLC B “Still Desirable”, yellow: HOLC C “Defnitely Declining”, red: HOLC D “Hazardous”). Backdrop shading: HOLC map, where colors refer to HOLC categories.

**Fig 12:**
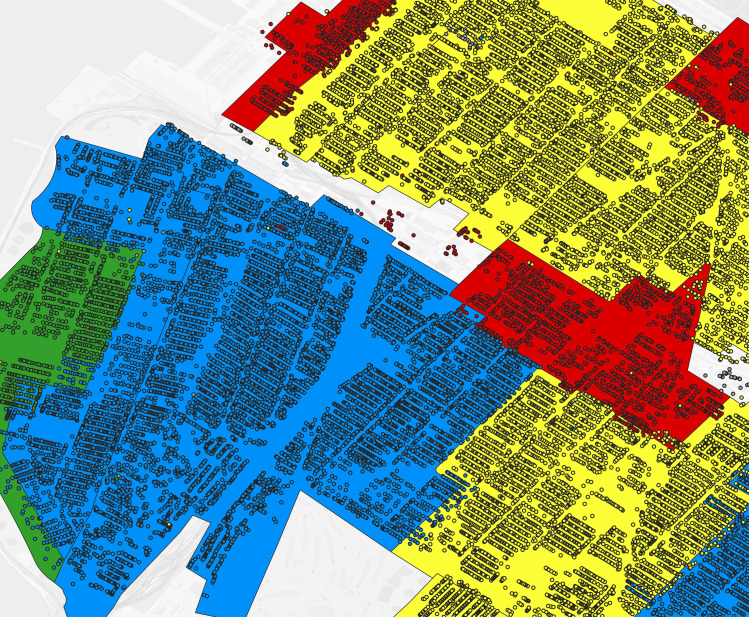
Brooklyn 1940 addresses overlaid with with 1930s HOLC map. Dot: one 1940 address where color refers to HOLC category assigned to the virtual enumeration district in Fig 11 (green: HOLC A “Best”, blue: HOLC B “Still Desirable”, yellow: HOLC C “Defnitely Declining”, red: HOLC D “Hazardous”), shaded polygons: HOLC map, where colors refer to HOLC categories.

Example maps of virtual 1940 enumeration districts merged with 1930s HOLC categories from a section of Brooklyn can be found in Figs 9–12.

## Discussion

### Principal results

We have created a new data resource and method for locating and generating virtual 1940 enumeration districts that can be used for future research for both redlining and for examining other health and geographic linkages in the 1940 census for urban areas. These linkages can be crucial for researchers who are exploring the impact of structural and social determinants of health and other contextual factors over a life course for present day health in older adults or in intergenerational studies. Our results show that the use of fast and relatively inexpensive (in terms of computational power, time, and money) geocoding and data processing algorithms can allow researchers to begin linking and analyzing historical geospatial data while waiting for more precise but intensive methods of digitizing and georeferencing historical documents.

We have also merged these 1940 enumeration districts with 1930s HOLC redlining areas. Our results from enumeration districts that have both real and virtual enumeration districts suggest that the vast majority of virtual enumeration districts are coded with HOLC categories the same way as real enumeration districts would be. These data can then be used to analyze how living in 1940 in different HOLC redlining categorizations are associated with health outcomes in the present day. The constructed “virtual” enumeration district maps can also be used with, and can be used to combine, any other data that either contain 1940 enumeration district identifier names–including longitudinal datasets like the Health and Retirement Study, Wisconsin Longitudinal Study, or Panel Study of Income Dynamics [21]–or any other mapped or georeferenced data [17,18,21,24,44–48].

### Limitations

Potential issues in enumeration district construction include the fact that real enumeration districts can have an element of concavity (e.g. agglomerating a group of city blocks into the shape of a “L”). Additionally, these virtual enumeration districts are not mutually exclusive, and do sometimes cross each other. Future work could include adapting a concave hull algorithm and/or Thiessen/Voronoi shapes in a computationally feasible manner. Virtual enumeration districts in general are smaller than real enumeration districts. Our method of mapping enumeration districts depends on the location of only residential addresses. Thus areas that have a low density of residences due to zoning for other land uses may result in size differences in non-random ways. This difference–in turn–may impact how HOLC category is discretized for each enumeration district (though our comparison of real to virtual enumeration districts in our dataset finds very few differences in HOLC category assignment). More broadly, the paucity of historical datasets of non-residential addresses may impede research into past commercial or industrial exposures important to longitudinal socioeconomic and health outcomes.

Missing enumeration districts are not likely to be all missing completely at random, since the available GRF dataset is by size of city population. As a result, we know we are missing enumeration districts for smaller incorporated cities (we covered 191 out of a total of 412 US cities in 1940 [52]). However, most of these locations do not have an existing HOLC map, so our enumeration districts cover the vast majority of the geographic space covered by HOLC maps.

Among cities that are covered, some portion of enumeration district missingness is random and some is likely not. Typographic errors in recording street names (or in how they were miscoded by modern day records digitizers) are likely to be missing completely at random. On the other hand, geocoding errors–where the OpenCage API returned to us an address that we deemed not likely to be within the bounds of the 1940 enumeration district–are possibly missing because roads and originally assigned houses were moved or demolished. Thus enumeration districts that were trimmed because they failed our minimum address radius test may be more likely to be areas that underwent drastic urban renewal and reconstruction throughout the late 20th and early 21st centuries, which in turn were more likely to be areas where politically marginalized communities resided in 1940. While an analysis of the features of trimmed enumeration districts is not possible at this time (since not knowing where they are, we cannot map area features or HOLC categories to them), this is an area where future work is necessary.

### Future directions

Other datasets that could potentially be linked or matched to the 1940 full-count census may also be used to tie together historical exposures with health throughout the 20th and early 21st centuries and into the present. For example, we plan to use the HOLC category of the enumeration district where an individual lived in the 1940 census with health data in older adults in the Health and Retirement Study to explore the association of redlining and health outcomes in those who were directly exposed to redlining.

Machine learning algorithms might also help us process and better draw enumeration districts. For data processing, deep learning image recognition [65,66] could be used in reading and processing enumeration district map data, as well as using optical character recognition to read in handwritten full count data (such as the now available 1950 full-count census data). Language models might also be used to better parse or correct misinterpreted atypical shorthand and address names in the full-count data (for example in places where an enumerator drew a line to indicate the address is a continuation from the page before, or where “41st ave” was reinterpreted by a modern day coder as “41 Saint Avenue”). Machine learning could also be used to draw constructed enumeration districts with higher precision and to identify erroneous geocodes automatically. We briefly experimented on a small subset of addresses with an isolation forest algorithm using address data geocoded from OpenCage that drew virtual enumeration districts that matched well with the Urban Transition Project’s enumeration districts, but the process was computationally intensive at scale.

## Conclusions

Our expansion of geocoded and mapped virtual 1940 census enumeration districts to 191 US cities and merging with 1930s HOLC redlining maps demonstrates that the use of open data sources, algorithmic processing, historical context, and multiple datasets can dramatically augment informational completeness for researchers using historical documents where more completely digitized documents are either not yet available or lost, even despite limitations that reduce fidelity to the original analog documents. Our method is flexible in that it can be used in tandem with either documents that have been digitized to a higher degree of informational completeness or with other methods that provide higher fidelity but take more time such as the 1940 enumeration district boundaries provided by the Urban Transition Project [43]. This in turn can allow researchers working on research critical to understanding health equity to include additional layers of contextual and structural factors. These factors can be applied even at the individual level and are important to understanding and characterizing social determinants of health and their antecedents, including relationships among health and social policy (including racism and government-sanctioned discrimination), housing, power hierarchies, geographic and social barriers, protective health factors, and exposures to hazards.

## Supporting information

S1 Fig Row headers and example rows from the Geographic Reference File. We used the Geographic Reference File to create our dataset. Individual id (histid) has been redacted.
(TIF)
