# Supplementary figures and images for: Using internet-assisted geocoding of 1940 census addresses to reconstruct enumeration districts for use with redlining and longitudinal health datasets
Source: PLOS Glob Public Health. 2025 Jan 15;5(1):e0004067. doi: 10.1371/journal.pgph.0004067 (PMC11734980; doi:10.1371/journal.pgph.0004067)

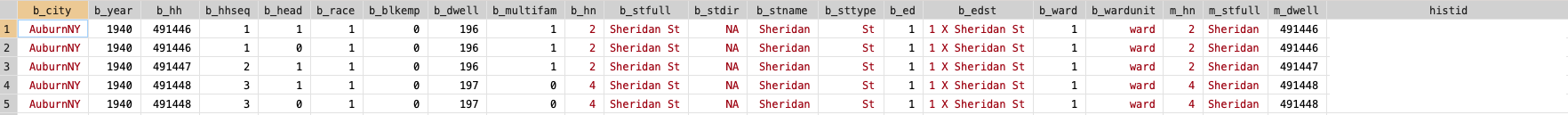

Supplement: S1 Fig — We used the Geographic Reference File to create our dataset. Individual id (histid) has been redacted. (TIF) [file pgph.0004067.s001.tif]
